# A model for cooperative gating of L-type Ca^2+^ channels and its effects on cardiac alternans dynamics

**DOI:** 10.1371/journal.pcbi.1005906

**Published:** 2018-01-16

**Authors:** Daisuke Sato, Rose E. Dixon, Luis F. Santana, Manuel F. Navedo

**Affiliations:** 1 Department of Pharmacology, University of California, Davis, Davis, CA, USA; 2 Department of Physiology & Membrane Biology, University of California, Davis, Davis, CA, USA; Universiteit Gent, BELGIUM

## Abstract

In ventricular myocytes, membrane depolarization during the action potential (AP) causes synchronous activation of multiple L-type Ca_V_1.2 channels (LTCCs), which trigger the release of calcium (Ca^2+^) from the sarcoplasmic reticulum (SR). This results in an increase in intracellular Ca^2+^ (Ca_i_) that initiates contraction. During *pulsus alternans*, cardiac contraction is unstable, going from weak to strong in successive beats despite a constant heart rate. These cardiac alternans can be caused by the instability of membrane potential (V_m_) due to steep AP duration (APD) restitution (V_m_-driven alternans), instability of Ca_i_ cycling (Ca^2+^-driven alternans), or both, and may be modulated by functional coupling between clustered Ca_V_1.2 (e.g. cooperative gating). Here, mathematical analysis and computational models were used to determine how changes in the strength of cooperative gating between LTCCs may impact membrane voltage and intracellular Ca^2+^ dynamics in the heart. We found that increasing the degree of coupling between LTCCs increases the amplitude of Ca^2+^ currents (I_CaL_) and prolongs AP duration (APD). Increased AP duration is known to promote cardiac alternans, a potentially arrhythmogenic substrate. In addition, our analysis shows that increasing the strength of cooperative activation of LTCCs makes the coupling of Ca^2+^ on the membrane voltage (Ca_i_→V_m_ coupling) more positive and destabilizes the V_m_-Ca_i_ dynamics for V_m_-driven alternans and Ca_i_-driven alternans, but not for quasiperiodic oscillation. These results suggest that cooperative gating of LTCCs may have a major impact on cardiac excitation-contraction coupling, not only by prolonging APD, but also by altering Ca_i_→V_m_ coupling and potentially promoting cardiac arrhythmias.

## Author summary

Recent experimental studies have suggested that clusters of L-type Ca_V_1.2 channels (LTCCs) can open and close in unison (i.e., cooperative or coupled gating) and that this gating modality may regulate excitation-contraction coupling in the heart. However, whether amplification of Ca^2+^ influx by cooperative gating of LTCCs promotes alternans is unknown. In this study, we developed a novel computational model of cooperative gating of LTCCs from experimental data. We incorporate the model into a physiologically detailed action potential (AP) model and investigated how changes in coupling strength of LTCCs may impact dynamics of AP and Ca^2+^ alternans. Our data suggest that increasing coupling strength of LTCCs prolongs AP duration and leads to Ca^2+^ overload. In addition, our theoretical and computational approaches elucidate that increasing coupling strength of LTCCs promotes positive Ca_i_→V_m_ coupling, which could lead to V_m_-driven and Ca^2+^-driven alternans.

## Introduction

L-type Ca_V_1.2 channels (LTCC) play a critical role in triggering cardiac muscle contraction during the action potential (i.e., excitation-contraction (EC) coupling) [1]. LTCCs are distributed in small clusters of about 10–12 [2–5] channels along the sarcolemma of these cells [1]. At the membrane potential reached during the plateau phase of the ventricular action potential (AP), LTCCs open, allowing Ca^2+^ ions to enter the cell. This Ca^2+^ signal is amplified via Ca^2+^-induced Ca^2+^ release through opening of ryanodine receptors (RyRs) from the sarcoplasmic reticulum (SR), which causes a cell-wide increase in Ca^2+^ that triggers cell contraction [6, 7].

Recent experimental studies [2, 8, 9, 28] have suggested that clusters of LTCCs can open and close in unison (i.e., cooperative or coupled gating). Functional coupling between LTCCs requires Ca^2+^ for the induction of physical interactions between adjacent channels that ultimately leads to amplification of Ca^2+^ influx. This suggests the intriguing hypothesis that cooperative gating of LTCCs may impact membrane voltage (V_m_) and intracellular Ca^2+^ (Ca_i_) cycling dynamics.

In cardiac myocytes, the dynamics of V_m_ are highly nonlinear. The LTCC current (I_CaL_) is one of the major currents, which determines the plateau membrane potential and regulates V_m_ dynamics. For example, a slower recovery of LTCC steepens the action potential duration (APD) restitution curve and promotes APD alternans [10–12]. Also, reactivation of LTCC during the plateau phase can cause early afterdepolarizations [13–15]. Furthermore, intracellular Ca^2+^ cycling also has its own nonlinear dynamics [12, 16]. In fact, AP clamp experiments showed that the unstable Ca_i_ cycling can induce Ca_i_ transient alternans without APD alternans [16]. The dynamics of Ca_i_ cycling is primary regulated by a steep SR Ca^2+^ release vs. SR Ca^2+^ load relationship [17–19] and thus RyR sensitization or Ca^2+^ overload often leads to Ca^2+^ alternans.

In this study, we generated a novel computational model of I_CaL_ based on experimental data and APs that incorporates cooperative gating of LTCCs and used it in physiologically detailed AP models to investigate the effects of varied degrees of LTCC coupling on Ca^2+^ entry, APD, and the likelihood of promoting voltage and Ca^2+^ alternans.

## Materials and methods

We developed two computational models. One is a totally stochastic model of cooperative gating (hereafter, we call it ‘stochastic model’). In this model, we simulated individual LTCCs described by a stochastic Markov model. Using this model, we investigated I_CaL_ properties by comparing with experimental observations. The other is a deterministic model based on the stochastic model (hereafter, we call it ‘ionic model’). Since simulation of the stochastic model is highly computationally intensive and thus time-consuming, we developed the ionic model to permit the investigation of steady state alternans within a practical time frame.

### Stochastic model

We used a physiologically detailed subcellular Ca^2+^ cycling model as in our previous studies [20–22], which is based on the rabbit ventricular myocyte model by Restrepo *et al*. [23]. Whereas the main structure of our model is similar to the Restrepo model, a key difference in our model is the open probability of RyRs. We reduced the number of RyRs opening during a spark from nearly 100 in the Restrepo model to only 5–10 to fit the experimental observation [20].

### Cell geometry

The dimension of this model is 121 μm × 25 μm × 11 μm and there are 19,305 (65×27×11) Ca^2+^ release units (CRUs). The separations of CRU are 1.84 μm and 0.9 μm in the longitudinal direction and transverse direction, respectively. Experimental observations indicate that each CRU contains about 10 LTCCs [24, 25]. Each CRU in this model contains at least 1 LTCC, but no more than 25. The cluster size obeys Gaussian distribution with a mean of 10 and standard deviation of 3. One CRU contains 100 RyRs, thus yielding ~1,930,500 RyRs in the cell. In each CRU, there are 5 compartments for Ca^2+^; cytosolic Ca^2+^ ([Ca^2+^]_i,_ *c*_*i*_ in equations), sub-membrane Ca^2+^ ([Ca^2+^]_s_, *c*_*s*_ in equations), cleft space Ca^2+^ ([Ca^2+^]_Cleft_, *c*_*p*_ in equations), network SR Ca^2+^ ([Ca^2+^]_SR_, *c*_*NSR*_ in equations), and junctional SR Ca^2+^ ([Ca^2+^]_JSR_, *c*_*JSR*_ in equations). CRUs are connected by Ca^2+^ diffusion in the cytosol and the network SR. The RyR is described by a four-state Markovian model and each RyR also opens stochastically depending on cleft space Ca^2+^ concentration ([Ca^2+^]_Cleft_) and junctional SR Ca^2+^ concentration ([Ca^2+^]_JSR_). SERCA pumps are distributed equally over the cell.

### Ca^2+^ cycling

Intracellular Ca^2+^ cycling is governed by the following equations:

$$\begin{array}{c} \frac{dc_{i}^{n}}{dt}=\beta_{i}\left(c_{i}^{n}\right)\left(I_{dsi}\frac{v_{s}}{v_{i}}-I_{up}-I_{TCi}+I_{ci}\right),\\ \frac{dc_{s}^{n}}{dt}=\beta_{s}\left(c_{s}^{n}\right)\left(I_{dps}\frac{v_{p}}{v_{s}}+I_{NCX}-I_{dsi}-I_{TCs}+I_{cs}-I_{cabk}-I_{slcap}\right),\\ \frac{dc_{p}^{n}}{dt}=\beta_{p}\left(c_{p}^{n}\right)\left(I_{r}+I_{CaL}-I_{dps}\right),\\ \frac{dc_{NSR}^{n}}{dt}=\left(I_{up}\frac{v_{i}}{v_{NSR}}-I_{tr}\frac{v_{JSR}}{v_{NSR}}+I_{cNSR}\right),\\ \frac{dc_{JSR}^{n}}{dt}=\beta_{JSR}\left(c_{JSR}^{n}\right)\left(I_{tr}-I_{r}\frac{v_{p}}{v_{JSR}}\right), \end{array}$$

where *v*_*i*_ is the local cytosolic volume, *v*_*s*_ is the local submembrane space volume, *v*_*p*_ is the local proximal space volume, *v*_*JSR*_ is the local JSR volume, *β*_*i*_ is the instantaneous buffer function for *c*_*i*_, *β*_*s*_ is the instantaneous buffer function for *c*_*s*_, *β*_*p*_ is the instantaneous buffer function for *c*_*p*_, *β*_*JSR*_ is the instantaneous buffer function for *c*_*JSR*_, *I*_TCi_ is time-dependent buffering to Troponin C for *c*_*i*_, *I*_TCs_ is time-dependent buffering to Troponin C for *c*_*s*_, *I*_*dsi*_ is the diffusive current between *c*_*s*_ and *c*_*i*_, *I*_*up*_ is the uptake current, *I*_*ci*_ is the nearest-neighbor diffusive current for *c*_*i*_, *I*_*cs*_ is the nearest-neighbor diffusive current for *c*_*s*_, *I*_*dps*_ is the diffusive current between *c*_*p*_ and *c*_*s*_, I_NCX_ is the sodium-calcium exchange current, *I*_*CaBk*_ is the background sarcolemmal membrane Ca flux, *I*_*SLCaP*_ is the sarcolemmal membrane Ca pump, *I*_*r*_ is the release current, *I*_*CaL*_ is the L-type Ca current, *I*_*tr*_ is the JSR refilling current, *I*_*cNSR*_ is the nearest-neighbor diffusive current for *c*_*NSR*_, superscript *n* shows the *n*-th compartment.

The diffusive currents between different compartments are the same as those previously employed by Restrepo *et al*. [23].

$$\begin{array}{l} I_{dsi}=\frac{c_{s}-c_{i}}{\tau_{si}}\\ \;I_{tr}=\frac{c_{NSR}-c_{JSR}}{\tau_{tr}}\\ I_{dps}=\frac{c_{p}-c_{s}}{\tau_{p}} \end{array}$$

### Cooperative gating of LTCCs and L-type Ca current

**Fig 1:**
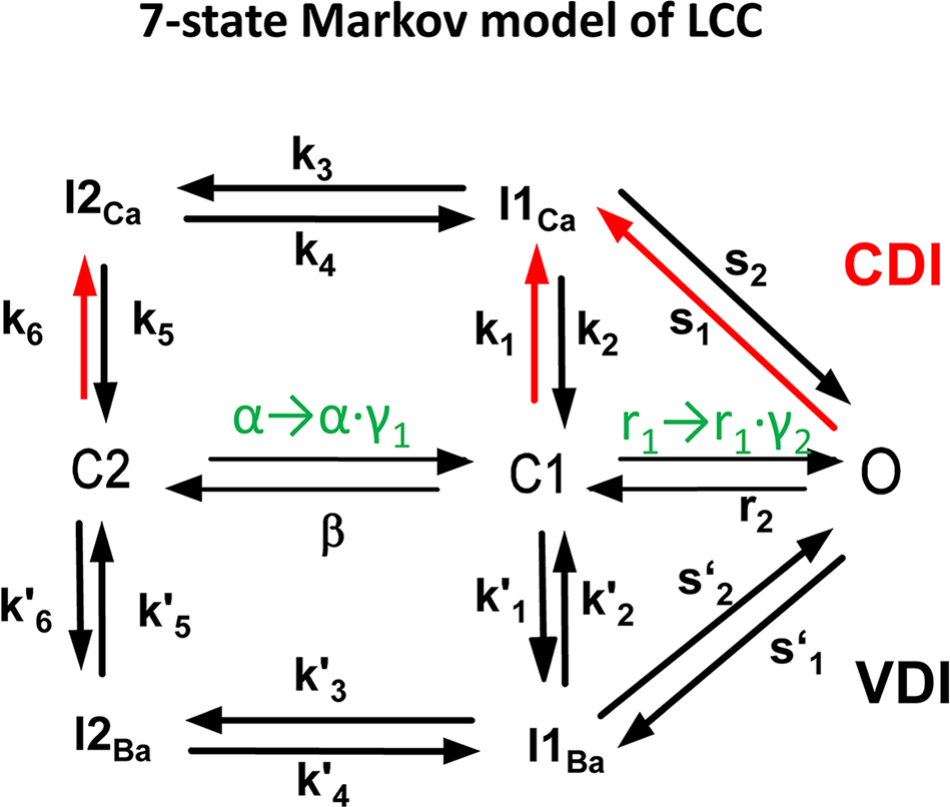
7-state Markov model of the L-type Ca channel. γ_1_ and γ_2_ are Ca^2+^ and the number of open channels dependent.

As stated above, each CRU contains between 1 and 25 LTCCs. *I*_*CaL*_ is described by

$$I_{CaL}=i_{Ca}N_{L}$$

$$i_{CaL}=4P_{Ca}zF\frac{{0.001\gamma }_{i}c_{p}{\;e}^{2z}-\gamma_{o}{\left[Ca\right]}_{O}}{e^{2z}-1}$$

where *i*_CaL_ is the single channel current, *N*_L_ is the number of open channels from 0 to 25, z = VF/(RT). LTCC activity is described by a Markov model with stochastic openings (Fig 1). *N*_L_ is determined by the number of open states of the Markov model within the CRU. Channel coupling and its [Ca^2+^]_Cleft_ dependence were incorporated in the LTCC model as follows. α in Fig 1 was replaced with α∙γ_1_ where

$$\gamma_{1}=1+w_{1}\frac{1}{1+exp\;(-15(N_{L}/3-po_{x}))}∙\frac{1}{1+exp\;(-1.0(c_{p}-cp_{x}))}$$

and r_1_ in Fig 1 was replaced with r_1_∙γ_2_ where

$$\gamma_{2}=1+w_{2}\frac{1}{1+exp\;(-15(N_{L}/3-po_{x}))}∙\frac{1}{1+\exp\;\left(-1.0(c_{p}-cp_{x})\right)}.$$

*po*_*x*_ and *cp*_*x*_ are parameters, which control the sensitivity of the coupling, *w*_*1*_ and *w*_*2*_ are the coupling strength. Other rates in Fig 1 are given by:

$$\begin{array}{l} \;\alpha =p_{o}^{\infty }/\tau_{po}\\ \;\beta =\frac{1-p_{o}^{\infty }}{\tau_{po}}\\ \;p_{o}^{\infty }=\frac{1}{1+e^{-V/8}}\\ \;s_{1}=0.02f(c_{p})\\ \;k_{1}=0.03f(c_{p})\\ \;s_{2}=s_{1}(k_{2}/k_{1})/(r_{1}/r_{2})\\ \;s_{2}^{\prime }=s_{1}^{\prime }(k_{2}^{\prime }/k_{1}^{\prime })/{(r}_{1}/r_{2})\\ f(c_{p})=\frac{1}{1+{\left(\tilde{c_{p}}/c_{p}\right)}^{3}}\\ \;k_{3}=\frac{e^{-\frac{\left(V+40\right)}{3}}}{3(1+e^{-\left(V+40\right)/3})}\\ \;k_{3}^{\prime }=k_{3}\\ \;k_{4}=k_{3}(\alpha /\beta )(k_{1}/k_{2})(k_{5}/k_{6})\\ \;k_{4}^{\prime }=k_{3}^{\prime }(\alpha /\beta )(k_{1}^{\prime }/k_{2}^{\prime })(k_{5}^{\prime }/k_{6}^{\prime })\\ \;k_{5}=\frac{1-P_{s}}{\tau_{Ca}}\\ \;k_{6}=f(c_{p})P_{s}/\tau_{Ca}\\ \;k_{5}^{\prime }=\frac{1-P_{s}}{\tau_{Ba}}\\ \;k_{6}^{\prime }=P_{s}/\tau_{Ba}\\ \;\tau_{Ca}=(R(V)-T_{Ca})P_{r}+T_{Ca}\\ \;\tau_{Ba}=(R(V)-T_{Ba})P_{r}+T_{Ba}\\ \;T_{Ca}=\frac{114}{1+{\left(c_{p}/\overset{-}{c_{p}}\right)}^{4}}\\ \;R(V)=10+4954e^{V/15.6}\\ \;P_{r}=\frac{e^{-\left(V+40\right)/4}}{1+e^{-\left(V+40\right)/4}}\\ \;P_{s}=\frac{e^{-(V+40)/11.32}}{1+e^{-(V+40)/11.32}} \end{array}$$

Note that the notations employed here are the same as those used by Mahajan *et al*. in [26]. A table of constants and the detailed formulation for other current fluxes to reproduce our results can be found in the supplemental material section.

### Ionic model

In order to investigate physiological and dynamic regulation of alternans by cooperative gating of LTCCs, we use the action potential (AP) and Ca_i_ cycling model of the ventricular myocyte by Shiferaw *et al*. [12] since we know which parameters control V_m_ and Ca^2+^ dynamics in this model.

The dynamics of membrane voltage (*V*_*m*_) are described by the equation:

$$\frac{dV_{m}}{dt}=-\frac{1}{C_{m}}(I_{ion}+I_{stim}),$$

where *I*_*ion*_ is the total membrane current density, *I*_*stim*_ is the stimulus current, and where *C*_*m*_ is the cell membrane capacitance. The total membrane current is given by:

$$I_{ion}=I_{Na}+I_{to}+I_{Kr}+I_{Ks}+I_{Kp}+I_{K1}+I_{NaCa}+I_{CaL},$$

where *I*_*Na*_ is the fast sodium current, *I*_*to*_ is the transient outward potassium current, *I*_*Kr*_ is the rapid component of the delayed rectifier potassium current, *I*_*Ks*_ is the slow component of the delayed rectifier potassium current, *I*_*Kp*_ is the plateau potassium current, *I*_*K*1_ is the inward rectifier potassium current, *I*_*NaCa*_ is the sodium-Ca^2+^ exchanger, and *I*_*CaL*_ is the L-type Ca^2+^ current.

Ca^2+^ cycling was modeled by following Shiferaw *et al*. [19]. This model describes Ca^2+^ released from the SR as a summation of local release fluxes distributed throughout the cell. The equations for Ca^2+^ cycling are:

$$\begin{array}{l} \;\frac{dc_{s}}{dt}=\frac{\beta_{s}v_{i}}{v_{s}}[J_{rel}-\frac{c_{s}-c_{i}}{\tau_{s}}+J_{Ca}+J_{NaCa}],\\ \;\frac{dc_{i}}{dt}=\beta_{i}[\frac{c_{s}-c_{i}}{\tau_{s}}-J_{up}],\\ \;\frac{dc_{j}}{dt}=-J_{rel}+J_{up},\\ \;\frac{dc_{j}^{\prime }}{dt}=\frac{c_{j}-c_{j}^{\prime }}{\tau_{a}},\\ \frac{dJ_{rel}}{dt}=gJ_{Ca}∙Q(c_{j}^{\prime })-\frac{J_{rel}}{\tau_{r}}, \end{array}$$

where *c*_*s*_, *c*_*i*_ and *c*_*j*_ are the average concentrations of free Ca^2+^ in a thin layer just below the cell membrane, in the cytosol, and the SR, with volumes *v*_*s*_, *v*_*i*_ and *v*_*sr*_ respectively. Here the SR volume includes both JSR and NSR. Also $c_{j}^{\prime }$ is the average JSR Ca^2+^ concentration within dyadic junctions in the whole cell. The factors *β*_*i*_ and *β*_*s*_ describe instantaneous buffering to Calmodulin, the SR membrane, and Troponin C.

All Ca fluxes are divided by *v*_*i*_ and have units of μM/ms, which can be converted to units of μA/μF using the conversion factor *nFv*_*i*_/*C*_*m*_, where *n* is the ionic charge of the charge carrier, and where *F* is Faraday's constant. Therefore, ionic fluxes can be converted to currents by:

$$I_{Ca}=-2\alpha J_{Ca},\;I_{NaCa}=\alpha J_{NaCa},$$

where *α* = *Fv*_*i*_/*C*_*m*_, and where the ion currents are in units of μA/μF.

### The L-type Ca^2+^ current flux (*J*_*CaL*_):

*J*_*CaL*_ is given by

$$J_{CaL}={-g}_{Ca}∙d∙f∙f_{Ca}∙i_{Ca},$$

where *g*_*Ca*_ is the maximum conductance of *J*_*CaL*_, *d* is activation, *f* is voltage-dependent inactivation and *f*_*Ca*_ is the Ca^2+^ dependent inactivation, *i*_Ca_ is the single channel current.

To replicate the Markov model shown above, cooperative gating and its Ca^2+^ dependence were incorporated in the LTCC model as follows:

$$\begin{array}{l} \frac{d(d)}{dt}=\alpha_{d}\gamma_{d}(1-d)-\beta_{d}d,\\ \;d_{\infty }=\frac{1}{1+\exp\left(\frac{-(V_{m}+5)}{6.24}\right)}\\ \alpha_{d}=\frac{d_{\infty }}{\tau_{d}}\\ \beta_{d}=\frac{1-d_{\infty }}{\tau_{d}} \end{array}$$

$$\gamma_{d}=1+0.01∙w∙\frac{1}{1+\exp\;\left(-15(po_{Ca}-po_{x})\right)}∙\frac{1}{1+\exp\;\left(-1.0(cs-cs_{x})\right)},$$

where γ_d_ is the coupling, which depends on the open probability of LTCC (*po*_*Ca*_ = *d* ∙ *f* ∙ *f*_*Ca*_) and submembrane [Ca^2+^] (*c*_*s*_), *po*_*x*_ and *cs*_*x*_ are parameters, which control the sensitivity of the coupling, *w* is the coupling strength.

Voltage-dependent inactivation and the Ca^2+^ dependent inactivation are given by

$$\begin{array}{l} \;\frac{df}{dt}=\frac{f_{\infty }-f}{\tau_{f}}\\ \;f_{\infty }=\frac{1}{1+\exp\left(\frac{V_{m}+35}{8.6}\right)}\\ \;\frac{df_{Ca}}{dt}=\frac{f_{Ca}^{\infty }-f_{Ca}}{\tau_{fca}}\\ \;f_{Ca}^{\infty }=\frac{1}{1+{\left(\frac{c_{s}}{\hat{c_{s}}}\right)}^{\gamma }} \end{array}$$

The single channel current is given by

$$i_{Ca}=\frac{4V_{m}F^{2}}{RT}\left(\frac{c_{s}e^{2a}-0.34{\left[Ca^{2+}\right]}_{O}}{e^{2a}-1}\right)$$

with *a* = *V*_*m*_*F*/*RT*.

We varied the recovery time constant (*τ*_*f*_) of the inactivation gate (*f*) of LTCCs to control the stability of the V_m_ system since it is known that the steepness of APD restitution is sensitive to it. In order to control the stability of the Ca^2+^ system, we varied the steepness of the slope (*u*) of the SR Ca^2+^ release function, which controls the sensitivity of release to SR load. The degree of Ca^2+^ dependent inactivation (γ) was varied to obtain positive Ca_i_→V_m_ coupling (γ = 0.7) and negative Ca_i_→V_m_ coupling (γ = 1.5).

Tables of constants and the detailed formulation for other current fluxes and buffers can be found in the supplemental material section. All programs were written in C++ and run on a 24-node High-Performance Computing cluster and Amazon Cloud Computing Services. An expanded section with all equations can be found in the supplemental material section. The C++ code is available via our website.

## Results and discussion

### Model of cooperative gating (Stochastic model)

We built a stochastic model of cooperative gating of LTCCs and incorporated this gating modality into the subcellular Ca^2+^ cycling model, which has realistic Ca^2+^ compartments and diffusion. In this model, LTCC and RyR activity depends on the V_m_, [Ca^2+^]_Cleft_, the degree of LTCC coupling within a cluster as well as [Ca^2+^]_Cleft_ and [Ca^2+^]_JSR_ (RyR).

**Fig 2:**
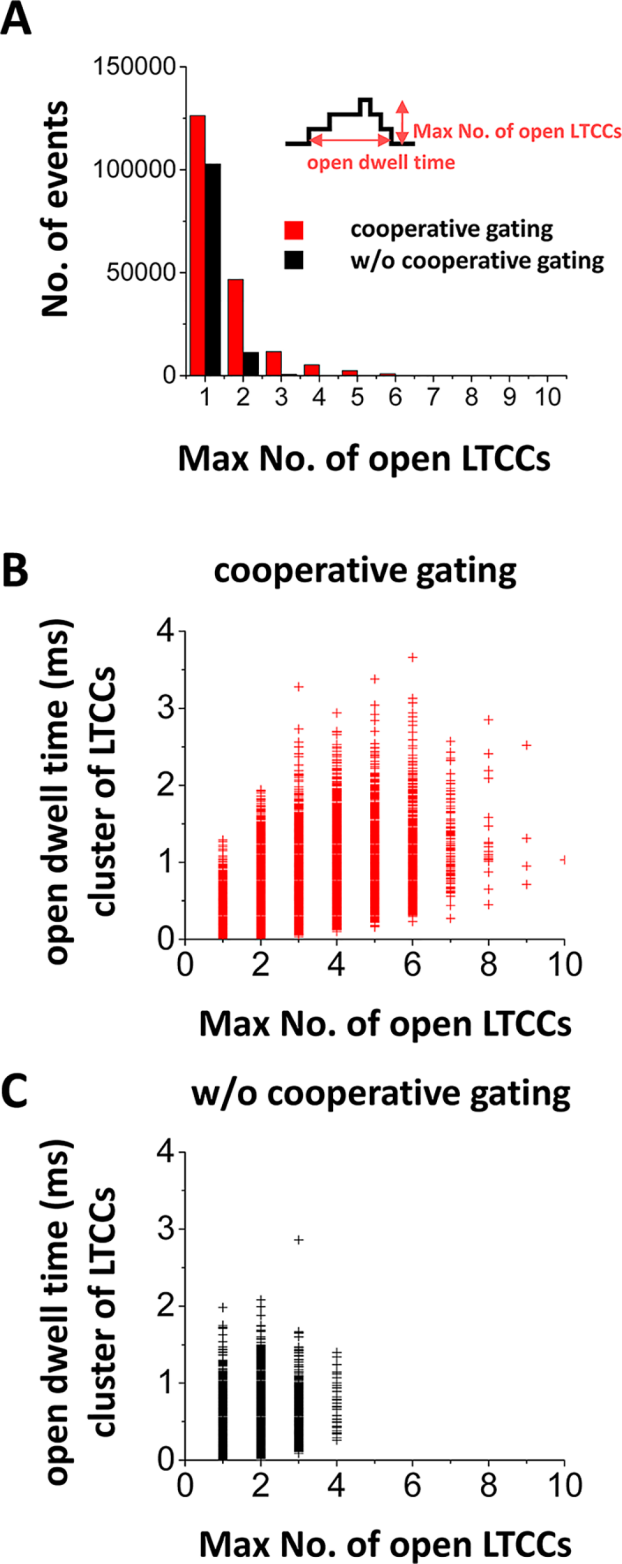
The stochastic model of cooperative gating of LTCCs. In this model, LTCCs and RyRs open stochastically. **(A)** The histogram of the maximum number of open LTCCs. Cooperative gating promotes simultaneous opening. **(B)** open dwell time of the cluster of LTCCs with coupling gating of LTCCs. **(C)** open dwell time of the cluster of LTCCs without coupling gating of LTCCs. In these simulations, the CRU contains 10 LTCCs.

**Fig 3:**
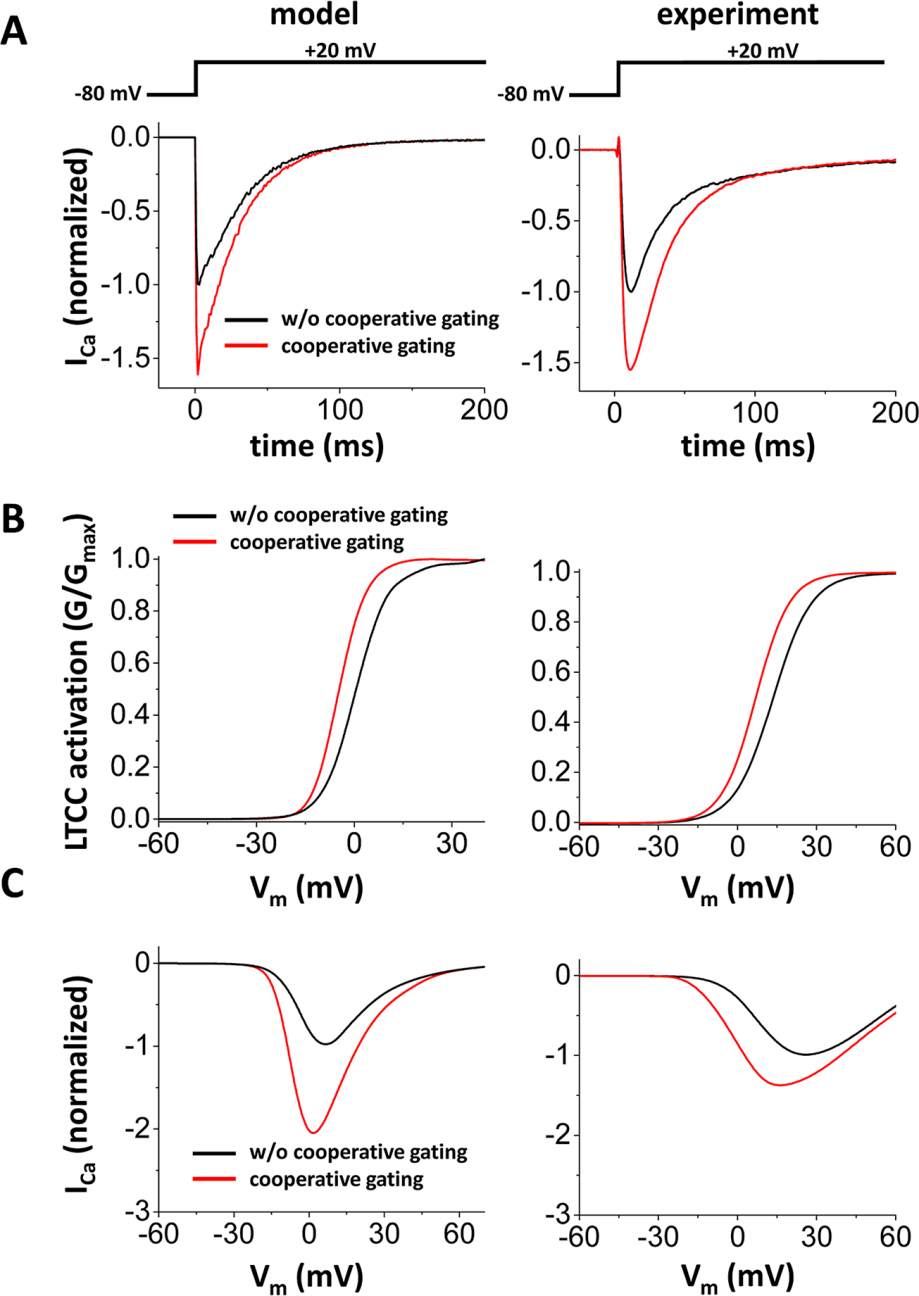
The stochastic model of cooperative gating of LTCCs (properties of I_CaL_). Left panels: Model results Right panels: Experimental results reconstructed from ref [9]**. (A)** I_CaL_ vs time when V_m_ is depolarized from -80 mV to +20 mV. **(B)** Activation curve of LTCC without cooperative gating (black) and with cooperative gating (red). **(C)** I-V curve of I_CaL_ without cooperative gating (black) and with cooperative gating (red).

Validation of the model involved the use of experimental data. When LTCCs are coupled, simultaneous opening events occurred more often (Fig 2A). Without cooperative gating, events of simultaneous opening of >2 channels are rare. On the other hand, with cooperative gating, events of simultaneous opening of 2 to 6 channels often occurred. Also, open dwell time of the LTCC cluster becomes longer with cooperative gating of LTCCs (Fig 2B vs 2C). We also measured the current-voltage relationship of I_CaL_ and the activation curve. To measure these curves, we used the same protocol used in the experimental study by Dixon *et al*. [9]. To be more specific, the membrane potential was depolarized from a holding potential of -80 mV to a specified test potential. Fig 3A shows one example of I_CaL_ vs time when V_m_ is depolarized from -80 mV to +20 mV. When cooperative gating is introduced, the peak of I_CaL_ was about 1.5 times larger than that of I_CaL_ without coupling. Cooperative gating of LTCCs shifted the activation curve to the left about 5 or 6 mV (Fig 3B) and nearly doubled the peak I_CaL_ (Fig 3C). Activation occurs at slightly lower voltage in the model. This discrepancy could be due to species differences as the model is built based on rabbit experiments [26] yet the experimental data collected by Dixon *et al*. was obtained from mouse cardiomyocytes [9]. Regardless of this, our *in silico* results are generally consistent with the previously reported experimental observations [9, 27]. Furthermore, they support the use of this model to examine the effects of LTCC coupling on Ca^2+^ entry, APD, and voltage and Ca^2+^ alternans.

**Fig 4:**
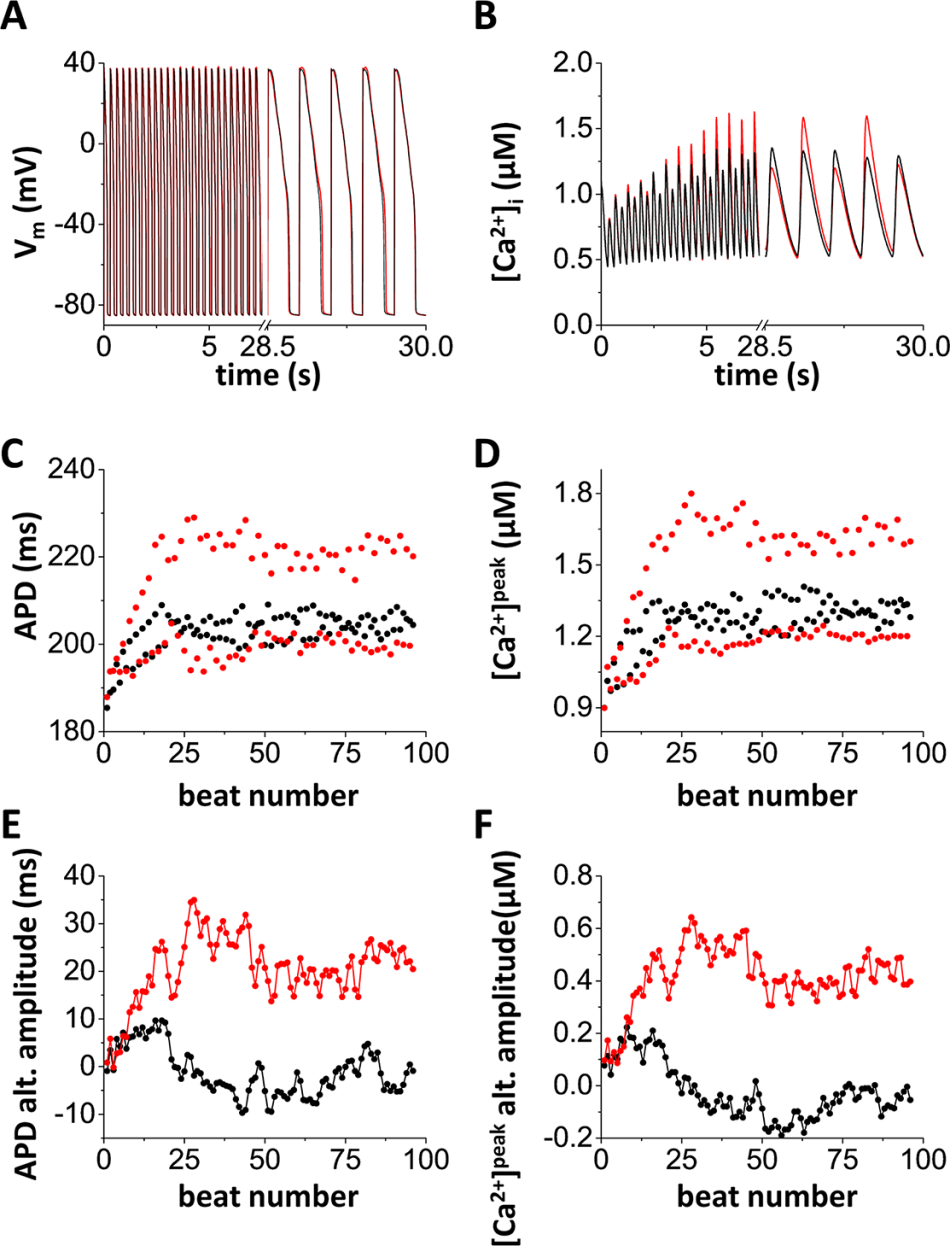
Cooperative gating promotes alternans. The cell with (red) /without (black) cooperative gating was paced at PCL = 300 ms. The initial conditions are the same in both cases. Alternans was developed within 100 beats only if cooperative gating was introduced. **(A)** The membrane potential vs time. **(B)** cytosolic [Ca^2+^] vs time. **(C)** APD vs the beat number. **(D)** peak cytosolic [Ca^2+^] vs the beat number. **(E)** APD alternans amplitude vs the beat number. **(C)** peak cytosolic [Ca^2+^] alternans amplitude vs the beat number.

To test if cooperative gating promotes alternans, the cell was paced with and without cooperative gating at fast rates. Fig 4 shows the development of alternans and its steady states. The cell was paced at pacing cycle length (PCL) = 300 ms. Without cooperative gating, alternans was not observed (black traces in each panel). However, when cooperative gating was introduced, alternans was developed within 100 beats (Fig 4A: voltage and Fig 4B: Ca^2+^). It reached the steady state after ~30 beats (Fig 4C & 4D). APD alternans amplitude (ΔAPD) is defined as

$$\Delta APD={\left(-1\right)}^{n}({APD}_{n+1}-{APD}_{n}).$$

Ca^2+^ transient alternans amplitude (ΔCa^2+^) is defined as

$$\Delta {Ca}^{2+}={\left(-1\right)}^{n}([{Ca}_{n+1}^{peak}+{Ca}_{n}^{peak}).$$

Without cooperative gating, alternans amplitudes fluctuate around zero. On the other hand, if cooperative gating is introduced, alternans amplitudes stay and fluctuate around certain values (Fig 4E & 4F). When the cell is paced at a faster rate (PCL = 290 ms), alternans occurred in both cases. However, APD and Ca^2+^ transient alternans amplitudes were much larger when LTCCs were coupled (S1 Fig). These results are consistent with experimental results [9]. Ca^2+^ transient alternans was observed when the cell was paced at PCL = 300 ms using a clamped AP waveform. This implies that Ca^2+^ cycling is unstable and contributes development of alternans.

### Functional effects of cooperative gating on alternans: Mathematical analysis

**Fig 5:**
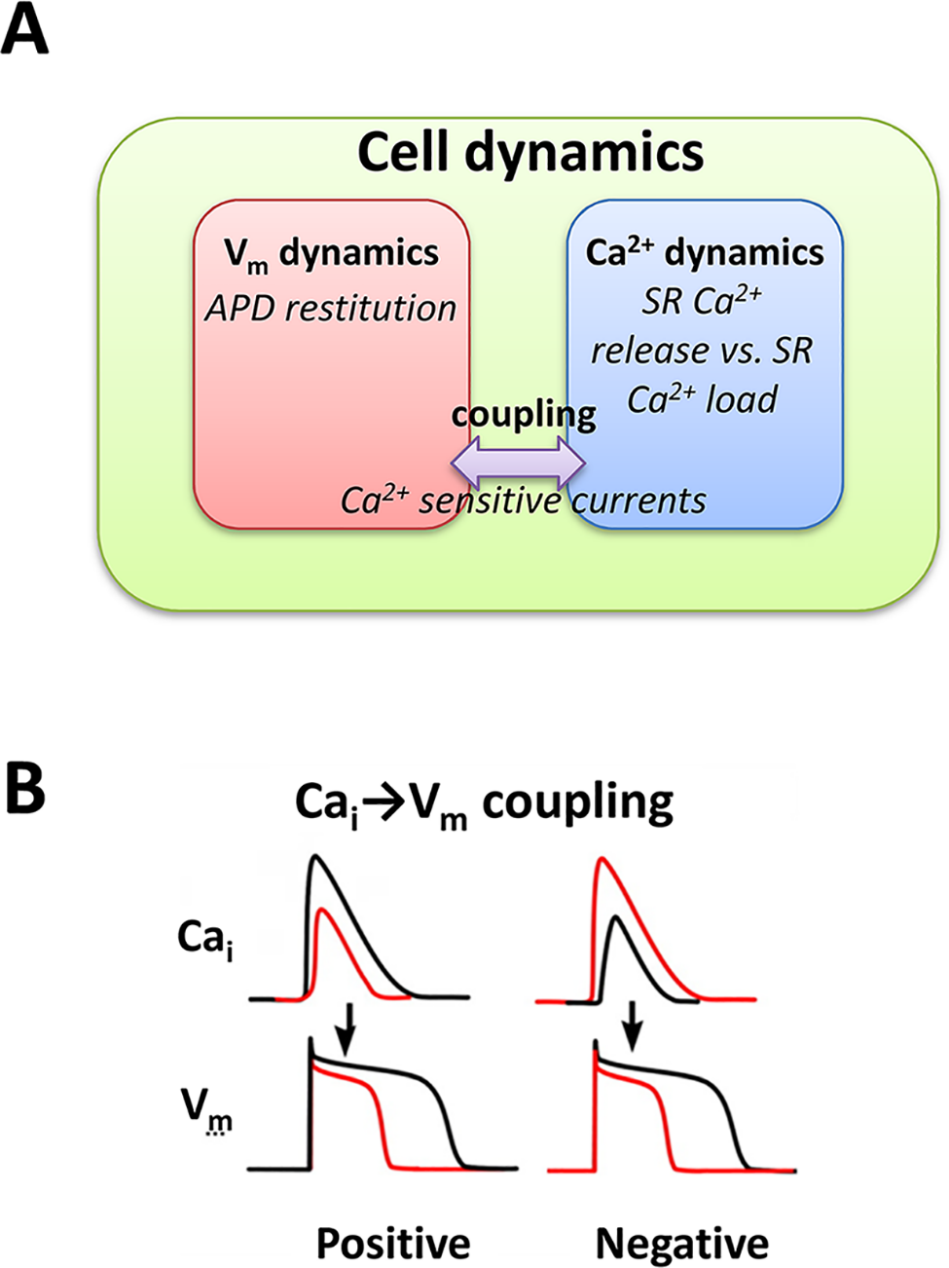
Cell dynamics and positive and negative Ca_i_→V_m_ coupling. **(A)** Coupled dynamics of V_m_ and Ca^2+^. The V_m_ dynamics is governed by APD restitution. Ca^2+^ dynamics is governed by SR Ca^2+^ release vs. SR Ca^2+^ load relationship. The V_m_ dynamics and Ca^2+^ dynamics are coupled via Ca^2+^-sensitive currents. **(B)** Positive and negative Ca_i_→V_m_ coupling.

**Fig 6:**
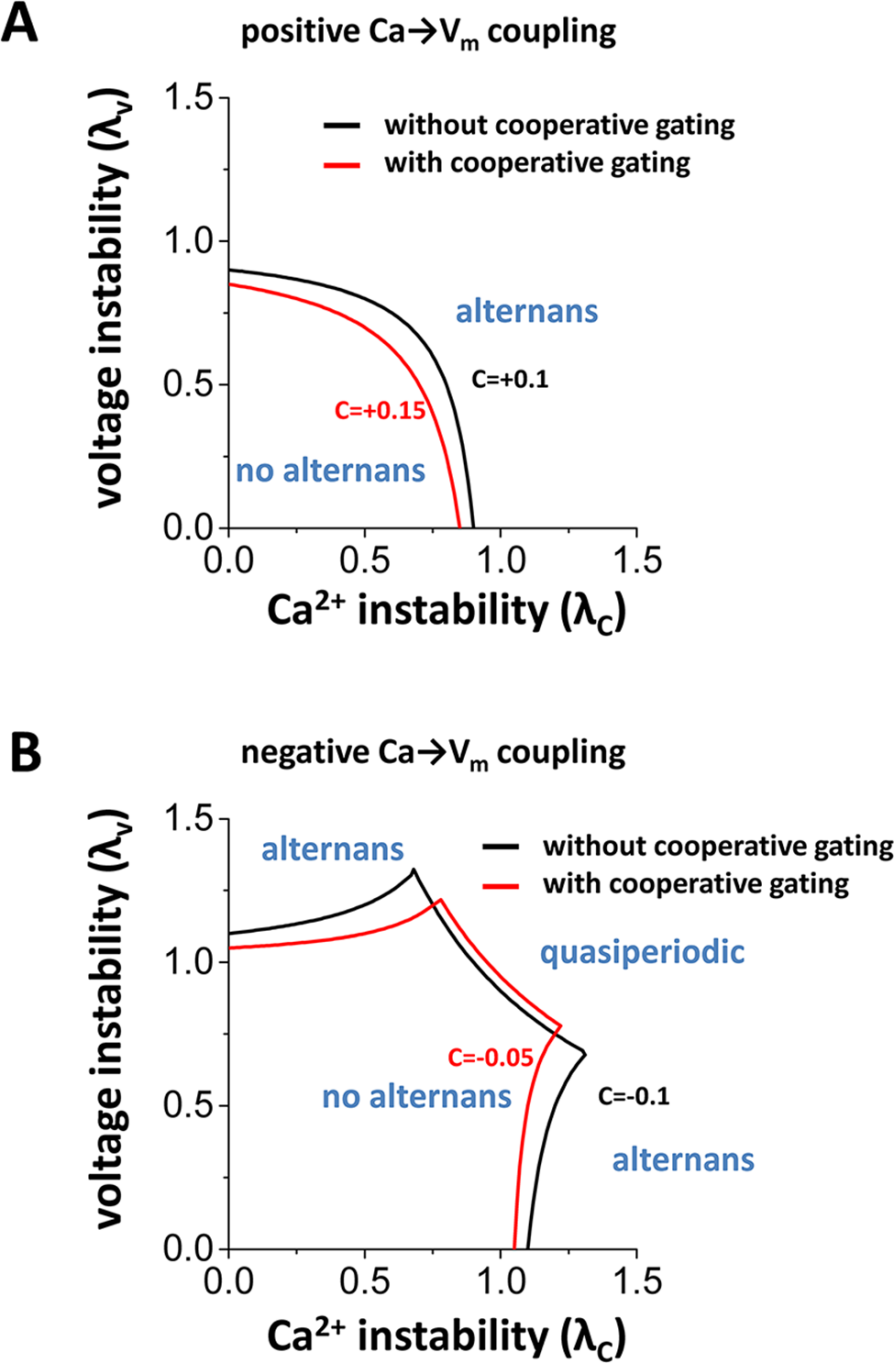
Theoretical analysis of the effects of coupled gating of LTCCs. **(A)** Stability boundary in the case of positive Ca_i_→V_m_ coupling. Black line *C* = 0.1 (without cooperative gating). Red line *C* = 0.15 (with cooperative gating). **(B)** Stability boundary in the case of negative Ca_i_→V_m_ coupling. Black line *C* = -0.1 (without cooperative gating). Red line *C* = -0.05 (with cooperative gating).

Alternans can be caused by instability of V_m_ due to steep APD restitution or instability of Ca_i_ cycling due to steep SR Ca^2+^ release vs. SR Ca^2+^ load relationship, or both [11, 12] (Fig 5A). The V_m_ dynamics and Ca^2+^ dynamics are coupled via Ca^2+^-sensitive currents such as I_CaL_ and the Na^+^-Ca^2+^ exchanger (NCX). When the Ca_i_ transient becomes larger, NCX prolongs the APD, whereas the I_CaL_ shortens the APD due to Ca^2+^-induced inactivation of the channel. Therefore, if NCX dominates, APD becomes longer as the Ca_i_ transient becomes larger. In our previous study [12], we defined this as positive coupling of Ca^2+^ on V_m_ (positive Ca_i_→V_m_ coupling, Fig 5B left). On the other hand, if I_CaL_ dominates, APD becomes shorter as the Ca^2+^ transient becomes larger. We defined this as negative coupling of Ca^2+^ on V_m_ (negative Ca_i_→V_m_ coupling, Fig 5B right). The stability of the coupled system is determined by the eigenvalues of a two dimensional map (see ref. [12] for details). The eigenvalues are:

$$\lambda_{\pm }=\frac{1}{2}(-\lambda_{v}-\lambda_{c}\pm \sqrt{{\left(\lambda_{c}-\lambda_{v}\right)}^{2}+4C}),$$

where λ_v_ is the eigenvalue associated with the map of the voltage system and λ_c_ is the eigenvalue associated with the map of the Ca^2+^ system, and *C* is coupling of Ca^2+^ on the membrane voltage. If V_m_ and Ca_i_ are uncoupled (*C* = 0), the system becomes unstable (i.e. alternans occurs) when λ_v_ or λ_c_ exceeds unity in absolute value. Black lines in Fig 6A and 6B show stability boundaries (|λ| = 1) when the Ca_i_→V_m_ coupling is positive (*C* = 0.1, Fig 6A) and negative (*C* = -0.1, Fig 6B) from our previous study [12].

It is also known that Ca^2+^ is required for the process of cooperative gating of LTCCs [2, 29]. Therefore, larger Ca^2+^ transients could tend to prolong APD due to increased strength of cooperative gating of LTCCs. In other words, cooperative gating of LTCCs may promote positive Ca_i_→V_m_ coupling. Consistent with this, red lines in Fig 6A and 6B show the stability boundary of alternans when Ca_i_→V_m_ coupling became more positive (C = 0.15, Fig 6A) and less negative (C = -0.05, Fig 6B). An increase in the strength of cooperative gating of LTCCs prolongs APD, which promotes steep APD restitution and increases Ca^2+^ influx, which may destabilize Ca^2+^ cycling. In addition, this analysis suggests that cooperative gating of LTCCs may destabilize the system (the stable areas became smaller) and promotes alternans except for the quasiperiodic regime.

### Functional effects of cooperative gating on alternans: Simulation (ionic model)

**Fig 7:**
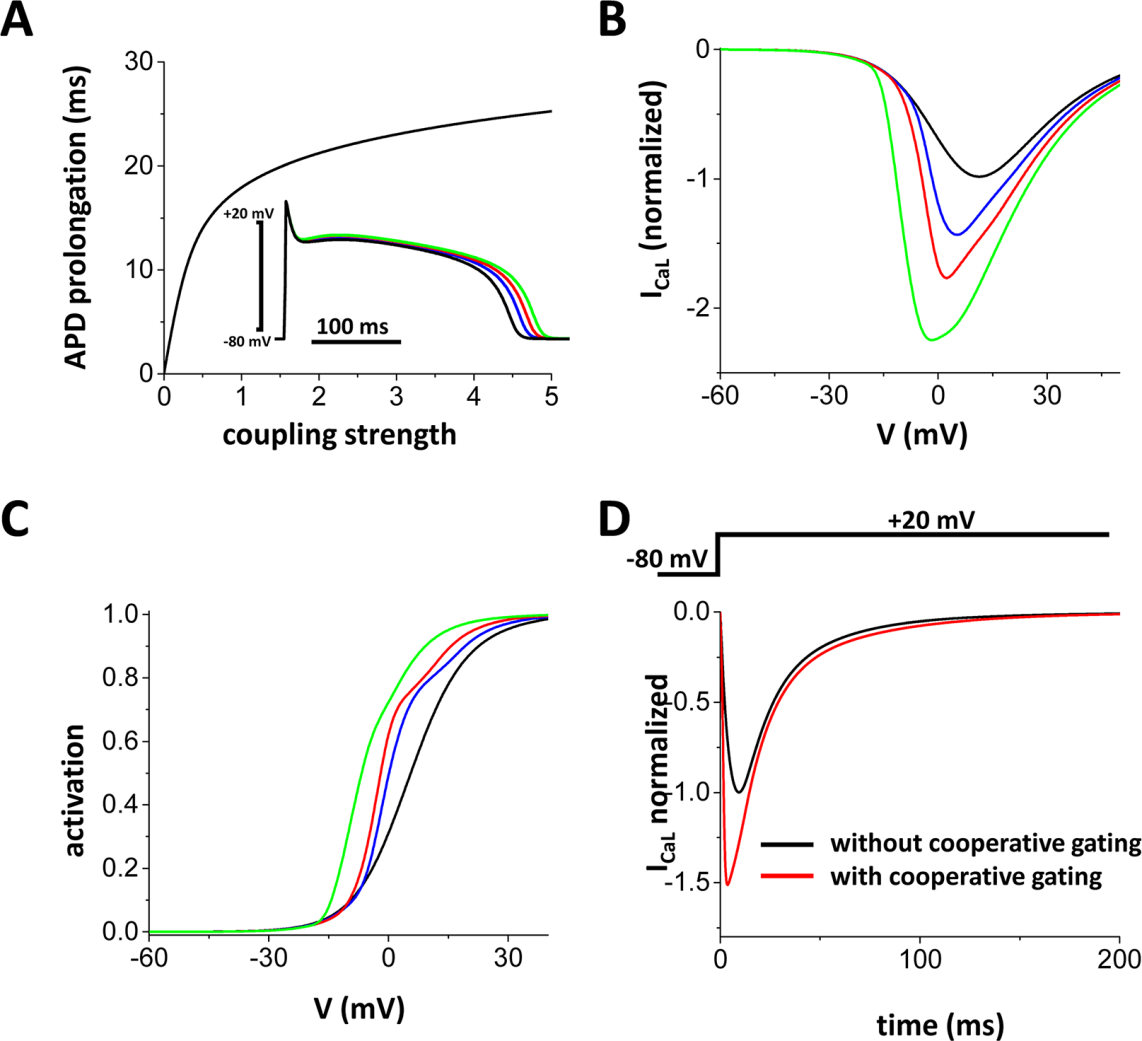
The deterministic model of cooperative gating of LTCCs. **(A)** Coupling gating of LTCCs prolongs APs. Inset: V_m_ vs time. Black: w = 0, Blue: w = 0.3, Red: w = 1, Green: w = 5. **(B)** I-V curve of I_CaL_. Black: w = 0, Blue: w = 0.3, Red: w = 1, Green: w = 5. **(C)** Activation curve of LTCC. Black: w = 0, Blue: w = 0.3, Red: w = 1, Green: w = 5. **(D)** I_CaL_ vs time when V_m_ is depolarized from -80 mV to +20 mV. w = 1.

To test our theoretical predictions above, we simulated alternans with the ionic model as described in the Methods section. Fig 7A shows that as the coupling strength became larger, APDs became longer. In this simulation, the cell was paced until it reaches the steady state. We chose parameters that do not cause alternans (*τ*_*f*_ = 45 ms, *u* = 3 s^-1^) without coupling (*w* = 0). Subsequently, the coupling strength was varied and the APD prolongation was measured. The inset shows the action potentials with *w* = 0.3 and 1.0. When the coupling strength is increased to 1.0, The peak of the current-voltage curve of I_CaL_ was almost doubled (Fig 7B). Increased coupling strength of LTCCs also shifts the activation curve to the left (Fig 7C). In Fig 7D, I_CaL_ vs time is shown when V_m_ is depolarized from -80 mV to 20 mV. Cooperative gating resulted in a 1.5-fold increase in peak I_CaL_. These data are consistent with the results with the stochastic model (Fig 3) and experimental observations [9, 27].

**Fig 8:**
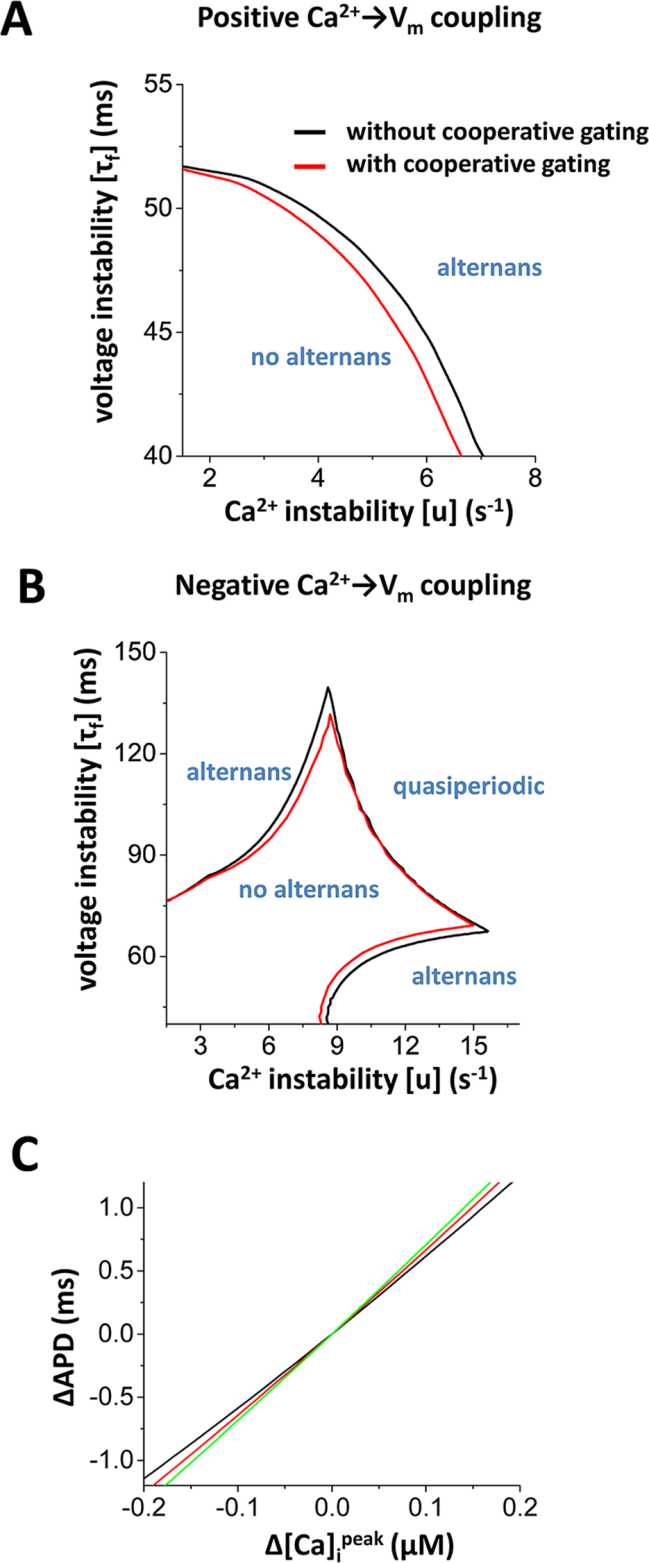
Coupling gating of LTCCs promotes positive Ca_i_→V_m_ coupling. **(A)** Stability boundary in the case of positive Ca_i_→V_m_ coupling. The coupling strength is 0.03. **(B)** Stability boundary in the case of negative Ca_i_→V_m_ coupling. The coupling strength is 0.03. **(C)** Coupling gating of LTCCs promotes positive Ca_i_→V_m_ coupling. The original Ca_i_→V_m_ coupling is positive. The slope became steeper with cooperative gating of LTCCs. The coupling strength is 0 (black), 0.05 (red), and 0.1 (green).

We also measured the stability boundary to test if increased strength of cooperative gating of LTCCs alters the stability as we predicted in the mathematical analysis (Fig 6). To investigate the effect of the change in channel cooperativity on alternans, we perturbed the cell by changing the coupling strength from *w* = 0 to *w* = 0.03. Fig 8A shows the stability boundary when Ca_i_→V_m_ coupling is positive and Fig 8B shows the stability boundary when Ca_i_→V_m_ coupling is negative. In both cases, V_m_-driven alternans and Ca^2+^-driven alternans are promoted with increased coupling strength of LTCCs. On the other hand, quasiperiodic oscillations are not affected. These results are consistent with the theoretical prediction (Fig 6A vs Fig 8A & Fig 6B vs Fig 8B). We note that this is not due to APD prolongation but due to Ca_i_→V_m_ coupling. In fact, unlike Fig 8B, simple prolongation of APD by the reduced potassium current stabilizes Ca^2+^-driven alternans when the coupling is negative (S3 Fig). If the coupling strength is within the physiological range, cooperative gating can change the sign of Ca_i_→V_m_ coupling from negative to positive (S4 Fig).

Fig 8C shows that as the coupling strength became larger, Ca_i_→V_m_ coupling became more positive. In this simulation, after reaching the steady state, initial [Ca^2+^]_SR_ was varied to change the amplitude of the Ca^2+^ transients and then the cell was paced once and the change in the APD was measured. The slope of Δ Ca^2+^ vs ΔAPD shows Ca_i_→V_m_ coupling. In Fig 8C, the slope became more positive as the coupling strength becomes larger, thus indicating that cooperative gating of LTCCs promotes positive Ca_i_→V_m_ coupling.

### Cooperative gating, excitation-contraction coupling, and arrhythmias

Cooperative gating of LTCCs facilitates synchronized opening of LTCCs, which may have a major impact on cardiac excitation-contraction coupling due to Ca^2+^ signal amplification. In this study, we built stochastic and deterministic computational models of cooperative gating of LTCCs and investigated how this gating modality may affect dynamics of the V_m_ and Ca_i_ cycling system, especially focusing on alternans, which is the arrhythmogenic substrate.

The novelty of our work is three-fold. *Firstly*, we model cooperative gating of LTCCs for the first time and add the complexity of this gating phenomenon to the existing models, bringing it more in-line with current thinking on Ca^2+^ signaling. We have thus generated a computational model encompassing cooperative gating of LTCCs, which has not been done before. *Secondly*, we model the effects of cooperative gating on alternans, finding that, in agreement with previously published experimental data [9], aberrant levels of cooperative gating can lead to Ca^2+^ alternans. Our theoretical and computational approaches suggest that increases in the strength of cooperative gating of LTCCs promotes positive Ca_i_→V_m_ coupling and thus promotes V_m_-driven and Ca-driven alternans. *Finally*, we confirmed that our model could reproduce experimental data, by specifically examining the effects of changes in the strength of cooperative gating of LTCCs on L-type Ca^2+^ currents. The degree of LTCC cooperativity can vary depending on physiological and pathological conditions. Our model provides an *in silico* means to explore the effects of LTCC cooperative gating under various conditions. In addition, Ca_i_→V_m_ coupling at the cellular level has been linked to mechanisms of spatially discordant alternans in tissue [30–32]. These findings underscore the importance of cooperative gating of LTCCs in excitation-contraction coupling and cardiac arrhythmias.

## Supporting information

S1 Fig The cell with (red) /without (black) cooperative gating was paced at PCL = 290 ms. The initial conditions are the same in both cases. Alternans was developed within 100 beats in both cases. **(A)** The membrane potential vs time. **(B)** cytosolic [Ca^2+^] vs time. **(C)** APD vs the beat number. **(D)** peak cytosolic [Ca^2+^] vs the beat number. **(E)** APD alternans amplitude vs the beat number. **(C)** peak cytosolic [Ca^2+^] alternans amplitude vs the beat number.
(TIFF)

S2 Fig The cell with cooperative gating was paced at PCL = 300 ms using a clamped AP waveform. Ca^2+^ transient alternans was observed. This demonstrate Ca^2+^ cycling is unstable and contributes development of alternans.
(TIFF)

S3 Fig Simple AP prolongation does not promote Ca-driven alternans when Ca_i_→V_m_ coupling is negative. G_Kr_ was reduced by 50%. (A) positive Ca_i_→V_m_ coupling (B) negative Ca_i_→V_m_ coupling.
(TIFF)

S4 Fig When the coupling strength is within physiological values (w = 0.3~1), negative Ca_i_→V_m_ coupling (black) became positive Ca_i_→V_m_ coupling (red). Black: w = 0, Red: w = 1.
(TIFF)
